# Investigating the Role of Carbohydrates Quantity and Quality in the Incidence of Metabolic Syndrome: A Two-Year Cohort Study: Short title: Fat and Sleep Quality

**DOI:** 10.31661/gmj.v15i.3984

**Published:** 2026-01-30

**Authors:** Melika Fallah, Azadeh Aminianfar, Awat Feizi, Peyman Adibi, Hamid Reza Rouhafza, Alireza Ani, Leila Azadbakht, Ahmad Esmaillzadeh

**Affiliations:** ^1^ Department of Community Nutrition, School of Nutritional Sciences and Dietetics, Tehran University of Medical Sciences, Tehran, Iran; ^2^ Research Center for Biochemistry and Nutrition in Metabolic Diseases, Kashan University of Medical Sciences, Kashan, Iran; ^3^ Department of Biostatistics and Epidemiology, School of Health, Isfahan University of Medical Sciences, Isfahan, Iran; ^4^ Integrative Functional Gastroentrology Research Center, Isfahan University of Medical Sciences, Isfahan, Iran; ^5^ Mental Health Department, Isfahan Cardiovascular Research Center, Isfahan University of Medical Sciences, Isfahan, Iran; ^6^ Department of Bioinformatics, Isfahan University of Medical Sciences, Isfahan, Iran.; ^7^ Department of Epidemiology, University of Groningen, University Medical Center Groningen, Groningen, Netherlands; ^8^ Diabetes Research Center, Endocrinology and Metabolism Clinical Sciences Institute, Tehran University of Medical Sciences, Tehran, Iran; ^9^ Obesity and Eating Habits Research Center, Endocrinology and Metabolism Molecular-Cellular Sciences Institute, Tehran University of Medical Sciences, Tehran, Iran; ^10^ Department of Community Nutrition, Isfahan University of Medical Sciences, Isfahan, Iran

**Keywords:** MetS, Cohort Study, Dietary Fiber, Whole Grain, Dietary CHOs

## Abstract

**Background:**

Metabolic syndrome (MetS) defines as a cluster of risk factors and is a global public health problem. Quality and quantity of carbohydrates (CHOs) intake plays a crucial role in the development of MetS. This study aimed to investigate the role of CHOs quantity and quality in relation to MetS.

**Materials and Methods:**

This prospective cohort study was conducted among healthy adults aged 18–65 years in Kerdabad, Isfahan, Iran. Participants (n=1904) were selected through a census-based sampling method. Dietary intake was assessed using a validated semi-quantitative food frequency questionnaire (FFQ). CHOs quantity and quality were evaluated using measures such as dietary glycemic index (DGI), dietary glycemic load (DGL), and fiber intake. MetS was defined by established clinical criteria ATPΙΙΙ and assessed annually over a two-year follow-up. Anthropometric measurements, blood pressure, and fasting blood samples were collected at baseline and yearly to monitor metabolic parameters. Statistical analyses included multivariate logistic regression models adjusting for potential confounders including age, sex, energy intake, physical activity, socioeconomic factors, and BMI.

**Results:**

Among 1904 participants (45% male; mean age: 39.6 ± 10.2 years; mean BMI: 27.1 ± 4.9 kg/m²), in fully adjusted models, none of the dietary CHOs quantity or quality indices were significantly associated with MetS during the two-year follow-up.

**Conclusion:**

quantity and quality of CHOs not significantly associated with MetS after adjusting for confounders. Although some trends indicated potential metabolic effects, these were not statistically significant. Further long-term research with more detailed dietary assessments is needed to clarify these relationships.

## Introduction

MetS is a complex cluster of physiological abnormalities, including abdominal obesity, dyslipidemia, elevated blood pressure and impaired fasting glucose [1]. It has become a major public health challenge worldwide [2]. This syndrome not only acts as a strong precursor for chronic non-communicable diseases such as type 2 diabetes, cardiovascular disease, and stroke [2], but also imposes significant costs on healthcare systems [3]. The increasing prevalence of MetS highlights the urgent need to identify modifiable risk factors, especially those related to lifestyle and diet [4]. Among macronutrients, CHOs play a pivotal role in providing energy and regulating metabolic processes [5]. Moreover, CHOs constitute the largest portion of dietary intake in many Asian countries compared to other macronutrients [6].

However, the importance of CHOs "quantity" versus "quality" has been widely debated [7]. Diets high in low-quality CHOs such as simple sugars and refined starches that cause rapid and high glycemic responses have been linked to increased risk of insulin resistance, chronic inflammation, and abdominal obesity [8]. In contrast, high-quality CHOs, including dietary fiber and complex CHOs found in whole grains, legumes, fruits, and vegetables, may have protective effects against MetS due to their lower glycemic index, higher fiber content, and beneficial influence on the gut microbiome [9].

Despite extensive research, comprehensive long-term studies examining both CHOs quantity and quality simultaneously in relation to MetS in specific populations remain limited. There are still uncertainties about the relative contributions of these aspects of CHOs consumption to the development of MetS, as well as the underlying mechanisms. Therefore, strong evidence from cohort studies with long-term follow-up is needed to clarify these complex relationships. The aim of this two-year cohort study is to comprehensively investigate the association between dietary CHOs quantity and quality and the risk of developing MetS in Iranian population. The findings may deepen our understanding of nutrition’s role in preventing MetS and help develop more precise and effective dietary recommendations to improve public health.

## Materials and Methods

### Study Design and Setting

This cohort study was conducted among healthy adults visiting health treatment centers in Kerdabad, Isfahan province, Iran. Kerdabad was selected as the study site due to its diverse population, representing a range of income levels and social status, thereby enhancing the potential generalizability of the findings to the broader Isfahan province population [10]. Households were sampled using a census-based method. Data collection occurred after obtaining informed consent from all participants [11]. This prospective cohort study commenced in September 2017. Anthropometric measurements, blood pressure, and fasting blood samples were collected at baseline and annually thereafter for two years to monitor metabolic parameters. Specifically, data collection occurred at three time points: at baseline (Year 0), and at approximately 12 and 24 months post-baseline (Years 1 and 2, respectively). The total follow-up duration for each participant was two years. All methods were conducted in accordance with relevant guidelines and regulations.

### Sample Size Calculation

The sample size was calculated to detect a significant relationship between diet and Mets, using the following formulas:


m' = \frac{\left[ c_{\alpha/2}\sqrt{(r+1)\bar{P}\bar{Q}} -
                            c_{1-\beta}\sqrt{rP_{1}Q_{1} + P_{2}Q_{2}} \right]^2}{r(P_{2} -
                            P_{1})^2}

m = \frac{m'}{4} \left[ 1 + \sqrt{1 + \frac{2(r+1)}{m' r |P_{2} -
                            P_{1}|}} \right]^2

P = (p1 + p2) / (1 + r)

Q1 = 1 - P1, Q2 = 1 - P2, Q = 1 - P

n1 = m

n2 = mr

n = n1 + n2

With a significance level (α) of 0.05 and a desired power (1 - β) of 80% (β = 0.2), the initial estimated sample size was 1246 individuals. Accounting for an anticipated dropout rate of 30%, the final target sample size was 1904 participants.

### Participants

**Figure-1:**
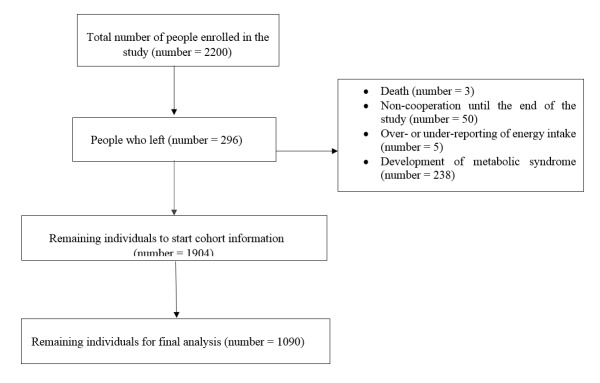
Figure 1. Flowchart of study participants

Inclusion criteria for participation were: (1) being an apparently healthy adult, (2) being aged between 18 and 65 years, and (3) being able to visit the local health center and complete the data collection questionnaire. Exclusion criteria were: (1) being a non-Iranian national, (2) being pregnant or breastfeeding, (3) Postmenopausal women, (4) having followed a specific dietary regimen within the preceding three months, and (5) having significant physical or mental disabilities that would impede the ability to visit a local health center or participate in data collection, (6) individuals who were on any chronic medication. Based on the initial sample size calculation, 1904 individuals were invited to participate. However, recruitment continued until a total of 2200 participants were enrolled. Subsequently, 58 participants were excluded due to mortality, lack of follow-through, reporting energy intake fell outside the plausible range, in addition 238 individuals were excluded from the study due to having MetS. Consequently, the final analysis included data from 1090 participants. A flowchart detailing participant enrollment and exclusions is presented in Figure-1.

### Data Collection

Dietary data were collected using a validated semi-quantitative FFQ comprising 106 food items in the Willett format. The FFQ’s validity and reliability have been previously established [12]. This instrument assesses the frequency of food consumption over the past year, providing an estimate of long-term dietary intake.

### Dietary Assessment

To convert FFQ data into nutrient values, the gram amount of each food item consumed by each participant was calculated based on reported frequency and portion sizes. Food items were then coded and entered into Nutritionist IV software (First DataBank, San Bruno, CA, USA), which was used to calculate the amounts and percentages of energy, CHOs, fiber intake and other nutrients consumed [13][14]CHOs quality was assessed by calculating DGI, DGL, whole grain/total CHOs intake, fiber/total CHOs intake [15][16][17]

### Calculation of DGI and DGL

Our methodology for calculating the DGI and DGL aligns with established practices, as detailed in previous studies [18][19][20]. Specifically, DGI was determined by summing the product of each food’s glycemic index and its available CHOs, then dividing this total by the aggregate available CHOs. Available CHOs were derived by subtracting the fiber content from the total CHOs for each food [19]. For DGL, we multiplied the glycemic index of the total diet by the total available CHOs and divided the result by 100, expressing it in grams per day.

### Assessment of MetS

In this study, participants with three or more of the following criteria identified as having MetS [21]: 1) Fasting blood glucose ≥ 100 mg/dLT, 2) Waist circumference ≥ 88 cm in women and ≥ 102 cm in men, 3) Systolic blood pressure ≥ 130 mmHg and diastolic blood pressure ≥ 85 mmHg, 4) Triglycerides ≥ 150 mg/dL, 5) HDL cholesterol < 40 mg/dL in men and < 50 mg/dL in women

### Study Follow-up

Following the initial cross-sectional phase, individuals diagnosed with MetS excluded from the study. Healthy participants followed prospectively for the development of MetS over two years. Blood samples will be collected annually to assess MetS-related factors. During each annual assessment, participants who met the criteria for incident MetS considered to have reached the study endpoint, and their participation conclude. Participants who remain free of MetS continue to be followed until the end of the two-year study period.

### Biochemical Assessments

For biochemical assessments, a 10 mL venous blood sample collected from each participant at baseline and at the end of each year for two years, following an overnight fast of at least 10 hours. Blood collected in sodium fluoride tubes. Samples transported in portable refrigerators maintained at 4-8°C and stored at -20°C in the central laboratory. Serum separated by centrifugation at 1500 g. Fasting blood glucose measured on the same day as sample collection using commercial kits employing the glucose oxidase/peroxidase method [22]. Serum lipid levels (total cholesterol, triglycerides, HDL cholesterol, and LDL cholesterol) measured using a BT3000 auto analyzer [22][23]. Total cholesterol measured using the cholesterol esterase-oxidase/peroxidase method [24]. High-density lipoprotein (HDL), low-density lipoprotein (LDL), and triglycerides determined using the glycerol kinase oxidase/peroxidase method [25].

All biochemical markers were measured using the Cobas c 311 analyzer (Roche Diagnostics, Mannheim, Germany). The corresponding commercial enzymatic kits were also supplied by Roche Diagnostics (Mannheim, Germany) and used according to the manufacturer’s instructions.

### Blood Pressure Measurement

Blood pressure measured using a mercury sphygmomanometer after participants rested for 15 minutes. With the participant seated, two measurements have been taken on the right arm, with a minimum interval of 5 minutes between measurements. The average of the two readings recorded as the participant’s blood pressure. Systolic blood pressure is defined as the first Korotkoff sound (phase 1), and diastolic blood pressure is defined as the disappearance of the sound (phase 5) during deflation of the cuff at a rate of 2-3 mmHg per second [26] .

### Anthropometric Measurements

Weight measured to the nearest 100g using a calibrated digital scale (SECA 831, Germany) with participants wearing minimal clothing [27]. Height measured with participants standing upright without shoes against a wall. Body mass index (BMI) then have been calculated using the standard formula [28]. Waist circumference measured at the narrowest part of the waist, over light clothing, without compression, during exhalation [29].

### Assessment of Other Variables

Information on other variables, such as age, sex, physical activity, education level, marital status, family size, and home ownership status, collected using a general questionnaire. This questionnaire assessed: Age: (in years), Sex: (Male/Female), Physical activity: Assessed using the GPAQ (General Physical Activity Questionnaire) and categorized into four levels: Never, Less than 1 hour, 1-3 hours, and More than 3 hours per week [30]. The reliability of this questionnaire was assessed using Cronbach’s alpha (α = 0.84) (60). Education level: (Higher or equal to diploma/Lower than diploma), Marital status: (Single, divorced, widowed/Married), Family size: (Less than or equal to four/Greater than four members), Home ownership: (Owner/Renter)

### Statistical Analysis

To report the general characteristics of participants with and without MetS, the independent t-test was used to compare continuous variables between two groups, and one-way ANOVA was used to compare continuous variables among more than two groups. These data are presented as mean ± standard deviation. To report categorical variables, the Chi-square test was used and presented as number (percentage).

To compare the dietary intakes and quantity and quality of CHOs of participants at the beginning of the study across tertiles of overall sleep quality and between individuals with and without MetS, analysis of covariance (ANCOVA) was used. All values are adjusted for age, sex, and energy intake, except for energy intake, which was adjusted only for age and sex. Values are presented as mean ± standard deviation.

The association between the quantity and quality of CHOs intake and the prevalence of MetS was examined using binary logistic regression analysis. Results were reported as odds ratios (ORs) with 95% confidence intervals (CIs). Three models were applied for adjustment. Model 1 was adjusted for age, sex, and total energy intake. In Model 2, additional adjustments were made for physical activity, marital status, socioeconomic status, and educational level. Finally, in Model 3, body mass index (BMI) was also included in the adjustments.

To examine the relative risk (95% CI) of developing MetS across tertiles of dietary fat and CHOs quantity and quality among the study participants, binary logistic regression was used. The results are presented as odds ratios and confidence intervals. The results were adjusted using three models: Model 1 was adjusted for age, sex, and energy intake; Model 2 was adjusted for the variables in Model 1 plus physical activity, marital status, socioeconomic status, and education; and Model 3 added body mass index (BMI) to the variables in Model 2 for adjustment.

## Result

**Table:** Table1. Comparison of the general characteristics of the participants with or without Mets at the beginning of the study

**Quantitative variable**	**Having metabolic syndrome **	**Having metabolic syndrome **	**p-value**
	**have**	**Have Not**	
		Mean±SD	
**Age (yaer) **	39/73±10/73	39/59±10/21	0/68
**BMI (kg.m ^2^** **)**	27/18±4/61	27/05±4/99	0/31
**Qualitative variable**		Number (%)	
**Classification of body mass index**			0/65
Low wight	9 (24/3%)	28 (75/7%)	
Normal weight	144 (23/5%)	469 (76/5%)	
Over weight	199 (26/4%)	554 (73/6%)	
obese	123 (25/7%)	355 (74/3%)	
**marital status**			0/003
married	394 (25/3%)	1164 (74/7%)	
widow	16 (51/6%)	15 (48/4%)	
divorced	4 (13/8%)	25 (86/2%)	
single	61 (23/3%)	201 (76/7%)	
**Education (yaer)**			0/32
12˂	150 (23/8%)	480 (76/2%)	
12≤	320 (25/9%)	916 (74/1%)	
**Socio economic status**			0/34
low	66 (28/7%)	164 (71/3%)	
moderate	237 (25/2%)	704 (74/8%)	
high	115 (23/6%)	372 (76/4%)	
**Physical activity**			0/67
never	114 (24/9%)	343 (75/1%)	
Less than one hour	120 (26/9%)	326 (73/1%)	
One to three hours	112 (25/6%)	326 (74/4%)	
More than three hour	104 (23/4%)	341 (76/6%)	

Values were obtained from one-way analysis of variance to compare quantitative variables among more than two groups. Independent t-test was used to compare quantitative variables between two groups. Chi-square test was used to compare the qualitative variables

**Table:** Table2. Comparison of dietary food intake of the participants among participants with or without MetS at the beginning of the study

**Having metabolic syndrome **	**Having metabolic syndrome **		**p-value**
**Energy (kcal.d) **	**have**	**Not have**	
**CHO (g.d) **	2456/03±58/26	2556/59±33/44	0/142
**Protein (g.d) **	320/11±3/32	318/96±1/90	0/76
**Fat (g.d) **	93/13±0/77	93/32±0/44	0/83
**PUFA(g.d)**	102/65±1/25	103/13±0/72	0/74
**MUFA(g.d) **	36/91±0/54	36/99±0/31	0/89
**SFA(g.d) **	27/26±0/38	27/39±0/22	0/76
**Tryptophan (mg.d) **	24/14±0/34	24/20±0/19	0/89
**Vitamin A (mg.d) **	792/12±12/03	784/95±6/90	0/60
**Vitamin C (mg.d) **	718/39±64/17	779/58±36/82	0/41
**Zinc (mg.d) **	116/17±2/7	115/28±1/58	0/78
**Calcium (mg.d) **	10/12±0/14	10/17±0/08	0/77
**Fe (mg.d) **	833/05±14/84	835/5±8/52	0/885
**Fiber intake (mg.d) **	23/28±0/53	23/771±0/309	0/440
**Total garin (g.d) **	15/91±0/19	15/899±0/110	0/963
**Animal fat (g.d) **	461/69±7/84	467/22±4/50	0/544
**Vegetable oil (g.d) **	10/83±0/40	10/46±0/23	0/43
**Dairy product (g.d) **	44/98±0/80	44/61±0/46	0/69
**Fruit (g.d) **	295/33±10/96	296/88±6/29	0/90
**Vegetables (g.d) **	370/22±12/33	370/06±7/07	0/99
**Legums (g.d) **	207/50±5/15	207/11±2/95	0/94
**Red meat (g.d) **	61/95±1/67	58/54±0/96	0/08
**White meat (g.d)**	68/98±1/72	68/67±0/91	0/88
**Fat (g.d) **	68/582±2/530	66/296±1/452	0/437

P-values are obtained from analysis of covariance. All values are adjusted for age, sex, and energy intake; Except for energy intake, which is only adjusted for age and sex

**Table:** Table3. Comparison of the quantity and quality of carbohydrate intake in individuals with and without metabolic syndrome

**variables**	**Having metabolic syndrome**		**P-value**
	**have**	**Not have**	
**Carbohydrate quantity**			
**Percentage of carbohydtrate**			
**Crude model**	51/07±0/44	50/63±0/25	0/40
**Adjusted model**	50/80±0/40	50/27±0/23	0/25
**Carbohydrate quality **			
**DGI**			
**Crude model**	61/63±0/28	61/52±0/16	0/74
**Adjusted model**	61/31±0/25	61/60±0/14	0/33
**DGL**			
**Crude model**	196/69±4/16	190/70±2/38	0/21
**Adjusted model**	189/13±4/88	193/96±2/83	0/16
**Dietary fiber intake**			
**Crude model**	15/89±0/21	15/97±0/12	0/75
**Adjusted model**	15/28±0/37	16/11±0/21	0/05
**Fiber/total carbohydrate intake **			
**Crude model**	0/05±0/001	0/05±0/000	0/29
**Adjusted model**	0/05±0/001	0/05±0/000	0/89
**Whole grain/total carbohydrate intake**			
**Crude model**	0/12±0/007	0/12±0/004	0/60
**Adjusted model**	0/12±0/008	0/12±0/008	0/32

P-values were obtained from analysis of covariance In the adjusted model, all values are adjusted for age, sex, energy intake, marital status, physical activity, education, body mass index, and socioeconomic status.

**Table:** Table4. Investigating the association between the quantity and quality of carbohydrate intake and the prevalence of metabolic syndrome among study participants

**Models**	**First tertile**	**Second tertile**	**Third tertile**	**P-trend**
**Percentage of carbohydrate intake** **Tertiles of **				
**OR (95%CI)**				
**crude Model **	1	1/18 (0/91, 1/53)	1/20 (0/93, 1/55)	0/16
**Model 1 **	1	1/11 (0/85, 1/45)	1/19 (0/91, 1/55)	0/19
**Model 2 **	1	1/05 (0/78, 1/41)	1/16 (0/87, 1/56)	0/28
**Model 3 **	1	1/05 (0/78, 1/41)	1/17 (0/87, 1/56)	0/28
**Tertiles of DGI**				
**OR (95%CI)**				
**crude Model **	1	1/11 (0/86, 1/43)	0/91 (0/70, 1/18)	0/51
**Model 1 **	1	1/19 (0/91, 1/54)	0/98 (0/75, 1/28)	0/90
**Model 2 **	1	1/24 (0/93, 1/66)	1/06 (0/79, 1/42)	0/66
**Model 3 **	1	1/26 (0/94, 1/67)	1/07 (0/80, 1/43)	0/63
**Tertiles of DGL**				
**OR (95%CI)**				
**crude Model **	1	1/10 (0/86, 1/42)	0/92 (0/71, 1/19)	0/4
**Model 1 **	1	1/31 (1, 1/73)	1/25 (0/90, 1/72)	0/15
**Model 2 **	1	1/28 (0/95, 1/74)	1/34 (0/94, 1/92)	0/09
**Model 3 **	1	1/28 (0/95, 1/74)	1/34 (0/94, 1/92)	0/09
**Tertiles of dietary fiber intake **				
**OR (95%CI)**				
**crude Model **	1	1/11 (0/86, 1/43)	0/94 (0/73, 1/22)	0/68
**Model 1 **	1	1/31 (0/99, 1/73)	1/39 (0/96, 2/02)	0/06
**Model 2 **	1	1/37 (1/008, 1/86)	1/40 (0/93, 2/01)	0/08
**Model 3 **	1	1/36 (1/004, 1/86)	1/39 (0/92, 2/09)	0/09
**Tertiles of fiber/total carbohydrate intake **				
**OR (95%CI)**				
**crude Model **	1	1/30 (1/01, 1/69)	1/19 (0/92, 1/54)	0/18
**Model 1 **	1	1/21 (0/93, 1/57)	1/04 (0/79, 1/36)	0/80
**Model 2 **	1	1/11 (0/83, 1/49)	1/04 (0/77, 1/40)	0/77
**Model 3 **	1	1/12 (0/83, 1/49)	1/04 (0/77, 1/40)	0/79
**Tertile of whole grain/total carbohydrate intake **				
**OR (95%CI)**				
**crude Model **	1	1/11 (0/85, 1/43)	1/09 (0/84, 1/41)	0/50
**Model 1 **	1	1/09 (0/84, 1/42)	1/05 (0/80, 1/37)	0/72
**Model 2 **	1	1/05 (0/78, 1/41)	0/98 (0/72, 1/31)	0/88
**Model 3 **	1	1/04 (0/77, 1/39)	0/97 (0/72, 1/31)	0/84

Obtained from binary regression analysis Model 1: Adjusted for age, sex, and energy intake Model 2: Additionally adjusted for physical activity, marital status, socioeconomic status, and education Model 3: Additionally adjusted for body mass index

**Table:** Table5. Relative risk (95% CI) of developing metabolic syndrome among the tertiles of carbohydrate intake quantity and quality among the study participants

**Models**	**First tertile **	**Second tertile **	**Third tertile **	**P-trend**
**Percentage of carbohydrate intake Tertiles of **				
**RR (95%CI)**				
**crude Model **	1	0/96 (0/67, 1/38)	1/05 (0/74, 1/49)	0/74
**Model 1 **	1	0/92 (0/63, 1/33)	1/05 (0/73, 1/50)	0/78
**Model 2 **	1	0/97 (0/66, 1/43)	0/88 (0/59, 1/31)	0/88
**Model 3 **	1	0/90 (0/60, 1/35)	1/02 (0/69, 1/50)	0/88
**Tertiles of DGI**				
**RR (95%CI)**				
**crude Model **	1	1/14 (0/80, 1/62)	1/11 (0/78, 1/59)	0/55
**Model 1 **	1	1/02 (0/83, 1/73)	1/20 (0/83, 1/73)	0/32
**Model 2 **	1	1/17 (0/78, 1/74)	1/19 (0/80, 1/77)	0/39
**Model 3 **	1	1/18 (079, 1/76)	1/19 (0/80, 1/77)	0/38
**Tertiles of DGL**				
**RR (95%CI)**				
**crude Model **	1	1/12 (0/79, 1/59)	0/96 (0/67, 1/37)	0/83
**Model 1 **	1	1/37 (0/93, 2)	1/48 (0/93, 2/43)	0/08
**Model 2 **	1	1/44 (0/95, 2/19)	1/56 (0/94, 2/57)	0/07
**Model 3 **	1	1/45 (0/95, 2/20)	1/57 (0/95, 2/59)	0/07
**Tertiles of fiber intake**				
**RR (95%CI)**				
**crude Model **	1	0/86 (0/61, 1/21)	0/75 (0/53, 1/07)	0/12
**Model 1 **	1	0/98 (0/67, 1/44)	0/99 (0/60, 1/65)	0/98
**Model 2 **	1	0/99 (0/65, 1/49)	1/02 (0/59, 1/77)	0/93
**Model 3 **	1	0/98 (0/65, 1/48)	1/01 (0/58, 1/75)	0/98
**Tertiles of fiber/total carbohydrate intake**				
**RR (95%CI)**				
**crude Model **	1	1/04 (0/73, 1/49)	0/99 (0/69, 1/40)	0/95
**Model 1 **	1	0/91 (0/63, 1/32)	0/85 (0/59, 1/23)	0/41
**Model 2 **	1	0/88 (0/59, 1/31)	0/82 (0/55, 1/22)	0/33
**Model 3 **	1	0/88 (0/59, 1/31)	0/81 (0/54, 1/21)	0/31
**Tertiles of whole grain/carbohydrate**				
**RR (95%CI)**				
**crude Model **	1	1/32 (0/92, 1/89)	1/18 (0/82, 1/69)	0/37
**Model 1 **	1	1/29 (0/89, 1/87)	1/13 (0/78, 1/64)	0/52
**Model 2 **	1	0/91 (0/60, 1/36)	1/15 (0/78, 1/69)	0/68
**Model 3 **	1	1/24 (0/83, 1/87)	1/08 (0/72, 1/62)	0/73

Obtained from binary regression analysis Model 1: Adjusted for age, sex, and energy intake Model 2: In addition to the previous factors, adjusted for physical activity, marital status, socioeconomic status, and education Model 3: In addition to the previous factors, adjusted for body mass index

1904 participants were included in this analysis within this analytic sample, 45% were male. The mean age of the study population was 39.63 ± 10.24 years, and the mean BMI was 27.08 ± 4.91 kg/m² respectively. Table-1 displays the baseline general characteristics of participants with and without Metabolic Syndrome (MetS). A notable difference emerged concerning marital status, a significantly higher proportion of married, divorced, and single individuals were found to be without MetS. Conversely, widowed individuals exhibited a significantly higher prevalence of MetS (P<0.05). This indicates a clear association between being widowed and a greater likelihood of having MetS, contrasting with other marital statuses. No other demographic characteristics showed statistically significant differences between the MetS and non-MetS groups (P>0.05).

A comparison of dietary food intake at the beginning of the study among individuals with and without MetS (Mets) is presented in Table-2. While no statistically significant differences were observed in general characteristics between the two groups, Table-2 details the dietary intake patterns. Although formal statistical testing did not reveal significant differences in most CHOs-related indices, the data presented in Table-2 should be examined for potential trends or clinical relevance, especially concerning the magnitude of any observed differences.

Table-3 presents a comparison of the quantity and quality of CHOs intake between individuals with and without MetS (Mets). Initially, our analysis revealed no statistically significant differences in most CHOs-related indices between the two groups. However, following adjustment for relevant confounders, a noteworthy observation emerged regarding dietary fiber intake as subjects without MetS exhibited a trend towards greater dietary fiber consumption (p = 0.05). While this finding approaches statistical significance, it underscores the importance of considering the potential magnitude and clinical relevance of such differences.

Table-4. Presents a comprehensive investigation of the association between the quantity and quality of CHOs intake and the prevalence of MetS among study participants. For dietary fiber intake, individuals in the second tertile, compared to the first tertile, showed no significant increase in prevalence in the crude model (95% CI: 0.73, 1.22; P-trend = 0.680) or the first adjusted model (95% CI: 0.96, 2.02; P-trend = 0.060). However, after adjusting for more confounders in the third model, a 37% increase in prevalence (95% CI: 1.080, 1.86; P-trend = 0.080) and in the fourth model, a 36% increase in prevalence (95% CI: 1.004, 1.86; P-trend = 0.090) was observed for MetS. However, the broad CIs suggest considerable uncertainty.

Regarding fiber/total CHOs intake, individuals in the second tertile, compared to the first tertile, showed a 30% increase in the prevalence of MetS in the crude model, which was statistically significant (95% CI: 1.01, 1.69; P-trend = 0.180). However, after adjusting for confounders, this significant association with MetS prevalence disappeared. In the third tertile of fiber/CHOs, compared to the first tertile, a 0.04% increase in the prevalence of MetS occurred, which was not statistically significant (95% CI: 0.77, 1.40; P-trend = 0.790). The wide confidence intervals across these measures underscore that while some trends are suggested, the data currently lack the precision for definitive conclusions regarding the impact of CHOs intake on MetS.

Relative risk (95% CI) of developing MetS among the tertiles of CHOs intake quantity and quality among the study participants summarizes in Table-5. After adjusting for potential confounders in the final model, no statistically significant associations were observed between CHOs intake and the risk of developing MetS, with a few exceptions. Individuals in the highest tertile of CHOs intake had a 0.02% higher risk compared to the first tertile (P-trend=0.88; 95% CI: 0.69, 1.50), while those in the highest tertile of DGI had a 19% higher risk compared to the lowest tertile (P-trend=0.38; 95% CI: 0.80, 1.77). Similarly, the third tertiles of DGL, dietary fiber intake, and the whole grains/total grains intake showed non-significant increased risks of 57% (P-trend=0.07; 95% CI: 0.95, 2.59), 0.01% (P-trend=0.98; 95% CI: 0.58, 1.75), and 0.08% (P-trend=0.73; 95% CI: 0.72, 1.62), respectively, compared to their corresponding first tertiles. The third tertile of the fiber/total CHOs intake showed a non-significant 19% decreased risk (P-trend=0.31; 95% CI: 0.54, 1.21). Crucially, all 95% CIs spanned the null value (1.00), indicating substantial uncertainty and no definitive conclusions.

## Discussion

This prospective cohort study investigated the association between quality and quantity of CHO intake and the prevalence and incidence MetS over a two-year period overall. By comparing individuals with and without MetS subjects not having mets had higher dietary fiber intake after adjusting for confounding factors but it was not statistically significant. For dietary fiber intake, the second tertile showed no significant difference compared to the first tertile in the simpler models, but after adjusting for more confounding factors, a relative increase in the prevalence of MetS was observed. In the third tertile compared to the first, none of the indicators showed a significant difference in the prevalence of MetS. After adjusting for confounders, there were no significant associations between CHO intake and incidence of MetS, although some non-significant trends were observed in the highest tertiles of CHO intake, DGI, DGL, fiber intake, and grain ratios.

### Total CHOs Intake and MetS

The lack of significant association between total CHOs intake and MetS in both cross-sectional and longitudinal analyses is consistent with several prior cohort [31][32][33]. These findings suggest that total CHOs intake alone may not be a strong predictor of metabolic health, particularly when the quality and source of are not differentiated [34] . From a mechanistic standpoint, while excess CHOs consumption especially from refined sources has been hypothesized to promote insulin resistance and dyslipidemia [35], these effects may be masked or counteracted when the CHOs intake comes from mixed or more balanced dietary patterns [36].

### Glycemic Index and Glycemic Load

Although higher tertiles of DGI and DGL were associated with increased risk and prevalence of MetS, these associations were not statistically significant after full adjustment. Previous studies have provided mixed results. For instance, one study showed significant associations between high GI/GL diets and increased cardiometabolic risk [37]. The discrepancy in our study may be due to differences in dietary patterns [37], lower dietary GL values overall [38]. Physiologically, high-GI and high-GL diets lead to greater postprandial glucose and insulin excursions, which over time may impair glucose metabolism, increase triglyceride synthesis, and reduce HDL cholesterol—all of which are components of MetS [38]. However, in populations with lower average GL, the cumulative metabolic burden may be insufficient to manifest as significant clinical risk [39] .

### Dietary Fiber and Fiber/CHOs Intake

In some of our study results, higher fiber intake was associated with MetS. This partially aligns with prior studies which demonstrated that higher fiber intake, particularly from whole plant sources, is inversely associated with MetS [40][41]. Fiber's beneficial role may stem from its effects on slowing glucose absorption [42], promoting satiety [43], modulating lipid metabolism [44], and reducing systemic inflammation [45]. The lack of statistical significance in our study could be attributed to relatively low fiber intake overall, or the dominance of low-fiber CHOs sources in the diet of the population studied.

### Whole Grain/total CHOs Intake

No significant relationship was observed between Whole grain/total CHOs intake and MetS outcomes. This contrasts with some other studies [46], which found strong inverse associations between whole grain intake and cardiometabolic risk [47].

This discrepancy could be due to limited consumption of whole grains in the study population, reducing the range of exposure and statistical power to detect an effect [48]. Additionally, differences in food processing methods, grain types, or cultural dietary practices may modify the impact of whole grains [49][50].

## Conclusion

In this two-year cohort study of Iranian adults, no statistically significant associations were found between CHOs intake, whether measured as total intake, DGI, DGL, dietary fiber, or CHOs quality ratios and the risk or prevalence of MetS. Although certain trends suggest possible metabolic effects of dietary CHOs quality, these did not reach statistical significance after full adjustment for confounding variables. Future long-term studies in diverse populations, with more granular dietary data and higher variability in CHOs sources, are necessary to better elucidate these complex relationships.

## Conflict of Interest

None of the authors had any personal or financial conflicts of interest.

## AI Disclosure Statement

During the preparation of this manuscript, the authors used ChatGPT, OpenAI company for language editing, grammar improvement, and liboberry.com for reference management. After its use, the authors thoroughly reviewed, verified, and revised all AI-assisted content to ensure accuracy and originality. The authors take full responsibility for the integrity and final content of the published article.
